# Mix-and-diffuse serial synchrotron crystallography

**DOI:** 10.1107/S2052252517013124

**Published:** 2017-10-09

**Authors:** Kenneth R. Beyerlein, Dennis Dierksmeyer, Valerio Mariani, Manuela Kuhn, Iosifina Sarrou, Angelica Ottaviano, Salah Awel, Juraj Knoska, Silje Fuglerud, Olof Jönsson, Stephan Stern, Max O. Wiedorn, Oleksandr Yefanov, Luigi Adriano, Richard Bean, Anja Burkhardt, Pontus Fischer, Michael Heymann, Daniel A. Horke, Katharina E. J. Jungnickel, Elena Kovaleva, Olga Lorbeer, Markus Metz, Jan Meyer, Andrew Morgan, Kanupriya Pande, Saravanan Panneerselvam, Carolin Seuring, Aleksandra Tolstikova, Julia Lieske, Steve Aplin, Manfred Roessle, Thomas A. White, Henry N. Chapman, Alke Meents, Dominik Oberthuer

**Affiliations:** a Center for Free-Electron Laser Science, Deutsches Elektronen-Synchrotron DESY, Notkestrasse 85, 22607 Hamburg, Germany; b Photon Science, Deutsches Elektronen-Synchrotron DESY, Hamburg, Germany; c Department of Physics, California State University, Northridge, California, USA; d The Hamburg Centre for Ultrafast Imaging, University of Hamburg, 22761 Hamburg, Germany; e Department of Physics, University of Hamburg, Luruper Chaussee 149, 22607 Hamburg, Germany; f Department of Physics, Norwegian University of Science and Technology, Trondheim, Norway; g Department of Physics and Astronomy, Uppsala University, Uppsala, Sweden; h European X-ray Free-Electron Laser Facility GmbH (XFEL), Schenefeld, Germany; i Department of Biochemistry, University of Oxford, Oxford, England; j SSRL, SLAC National Accelerator Laboratory, Menlo Park, California, USA; k Fachhochschule Lübeck, Lübeck, Germany

**Keywords:** drug discovery, protein structure, X-ray crystallography, serial crystallography, time-resolved studies, lysozyme

## Abstract

Unravelling the interaction of biological macromolecules with ligands and substrates at high spatial and temporal resolution remains a major challenge in structural biology. The development of serial crystallography methods at X-ray free-electron lasers and subsequently at synchrotron light sources allows new approaches to tackle this challenge. Here, a new polyimide tape drive designed for mix-and-diffuse serial crystallography experiments is reported. The structure of lysozyme bound by the competitive inhibitor chitotriose was determined using this device in combination with microfluidic mixers. The electron densities obtained from mixing times of 2 and 50 s show clear binding of chitotriose to the enzyme at a high level of detail. The success of this approach shows the potential for high-throughput drug screening and even structural enzymology on short timescales at bright synchrotron light sources.

## Abstract 2

The structure of chitotriose bound to lysozyme after mixing times of 2 and 50 s was determined using a polyimide tape-drive device for mix-and-diffuse serial crystallography at a synchrotron light source.

## 1. Introduction   

The structural information gathered from X-ray crystallo­graphic studies of proteins is incorporated into many stages of drug development (Congreve *et al.*, 2005 ▸; Blundell, 2017 ▸). For example, cancer, diabetes, inflammation and cardiovascular disease drugs that target kinase proteins have been developed by studying their binding sites (Parang & Sun, 2004 ▸), and anti-influenza drugs have been derived from the structure of neuraminidase (Varghese, 1999 ▸). More recently, the discovery of a new allosteric binding site in CCR9, where the ligand binds to the side of this chemokine receptor, is being explored as a new possible target for other G-protein coupled receptors (GPCRs; Oswald *et al.*, 2016 ▸). X-ray crystallographic fragment-screening studies have also shown the binding of small molecules that have an affinity that is too low to be detected by chemical assays (Schiebel *et al.*, 2016 ▸; Patel *et al.*, 2014 ▸; Erlanson *et al.*, 2016 ▸). Nonetheless, long-standing bottlenecks of solving ligand-bound protein structures include (i) time-consuming screenings of substrate co-crystallization or soaking conditions to produce stable crystals; (ii) deviations from the native protein structure induced by cryogenic temperatures; and (iii) the lack of fast sample-exchange systems for efficient data collection.

Serial crystallography offers a high-throughput platform to overcome these bottlenecks. This method entails using a Bragg intensity set merged from many snapshot diffraction patterns of individual protein microcrystals to solve the protein structure (Chapman *et al.*, 2011 ▸; Boutet *et al.*, 2012 ▸). The change in paradigm of this approach allows the total scattering signal per crystal to be reduced many thousands of times, enabling measurements from small crystals (Gati *et al.*, 2017 ▸) without the need for cryogenic cooling. Its use with the brilliant femtosecond pulses of an X-ray free-electron laser has led to a number of GPCR structures (Liu *et al.*, 2013 ▸; Zhang *et al.*, 2015 ▸; Kang *et al.*, 2015 ▸), *de novo* phasing (Barends *et al.*, 2014 ▸; Yamashita *et al.*, 2015 ▸; Colletier *et al.*, 2016 ▸) and the study of fast light-induced dynamics of photoactive proteins (Tenboer *et al.*, 2014 ▸; Barends *et al.*, 2015 ▸; Nango *et al.*, 2016 ▸; Pande *et al.*, 2016 ▸).

However, most biological macromolecules are not triggered by light. Time-resolved studies of enzymatic reactions thus require different triggering methods, such as the introduction of photo-caged compounds or optogenetic methods (Moffat, 2014 ▸), both resulting in artificially light-active proteins. It was also proposed to use temperature jumps or THz-radiation to investigate protein dynamics (Neutze & Moffat, 2012 ▸; Moffat, 2014 ▸). Another approach is to simply mix one or many crystals of a biological macromolecule with a ligand or substrate (chemical triggering as opposed to physical triggering) (Schmidt, 2013 ▸). This was demonstrated in the past for protein crystals using flow-cells (Hajdu *et al.*, 1987 ▸). The further development of sample delivery methods (Calvey *et al.*, 2016 ▸; Oberthuer *et al.*, 2017 ▸) for short mixing times, and the use of very small crystals in serial crystallography leading to short ligand diffusion times into those crystals (Schmidt, 2013 ▸), enabled the first successful mix-and-diffuse experiments at the LINAC Coherent Light Source (LCLS) (Stagno *et al.*, 2016 ▸; Kupitz *et al.*, 2017 ▸), with the aim of unraveling dynamics of the respective biological macromolecules under investigation. Such continuous room-temperature sample delivery also avoids stopped-flow or freeze-trapping experiments in combination with heavy mutations of the native protein that have previously been used to study long-lived intermediates (Stoddard, 1996 ▸; Schlichting & Goody, 1997 ▸).

Serial crystallography has also been demonstrated at microfocus beamlines of third generation synchrotron sources using exposure times of a few milliseconds (Stellato *et al.*, 2014 ▸; Botha *et al.*, 2015 ▸; Nogly *et al.*, 2015 ▸; Martin-Garcia *et al.*, 2017 ▸). This exposure time matches the expected diffusion time of molecules in protein microcrystals (Schmidt, 2013 ▸), and might be suitable in many cases for fragment-based drug screening and time-resolved structural enzymology studies. As a first step in this direction, we investigated chitotriose binding to hen egg-white lysozyme *via* mix-and-diffuse serial crystallography at a synchrotron source.

Chitotriose (*N*,*N*′,*N*′′-triacetylchitotriose; CTO) is a natural product that competitively inhibits lysozyme (Johnson, 1998 ▸). Lysozymes degrade bacterial cell walls by cleaving the 1,4-β-linkages between *N*-acetylmuramic acid and *N*-acetyl-d-glucosamine (GlcNAc) in peptidoglycan (Blake *et al.*, 1967 ▸). In addition, some lysozymes, including that from hen egg white, can cleave between GlcNAc residues in chitodextrins such as chitin. The cleft-like polysaccharide-binding site of lysozyme can accommodate up to six GlcNAc residues. Enzymatic cleavage (Cheetham *et al.*, 1992 ▸; Vocadlo *et al.*, 2001 ▸) occurs at the linkage between the GlcNAc residues occupying subsites *D* and *E* (Maenaka *et al.*, 1995 ▸; Von Dreele, 2005 ▸); consequently, pentasaccharides or longer polysaccharides are preferably processed. Shorter saccharides such as chitotriose, chitobiose or monomeric GlcNAc still bind with reasonable affinity to lysozyme, but take longer to be released, making them a natural competitive inhibitor for structural studies (Blake *et al.*, 1967 ▸; Cheetham *et al.*, 1992 ▸; Hadfield *et al.*, 1994 ▸; Maenaka *et al.*, 1995 ▸; Vocadlo *et al.*, 2001 ▸; Von Dreele, 2005 ▸).

## 2. Experimental procedure   

The lysozyme microcrystals used in this study were grown by the rapid-mixing batch method (Stellato *et al.*, 2014 ▸). Crystals with sizes of between 6 and 8 µm in diameter were obtained by adding three parts of precipitant [1 *M* NaCl, 40%(*v*/*v*) ethylene glycol, 15%(*w*/*v*) PEG 4000, 50 m*M* acetate buffer pH 3.5 filtered through a 450 nm filter] to one part of lysozyme (Sigma–Aldrich; dissolved to 126 mg ml^−1^ in 50 m*M* acetate buffer pH 3.5 and filtered through a 100 nm filter) at 1°C (ThermoStat C, Eppendorf, Germany). The resulting mixture was immediately subjected to rapid mixing and incubated for 30 min at 1°C. Shortly before the X-ray measurements, the suspension of microcrystals was centrifuged at 2000*g* and the supernatant was removed to achieve a twofold higher concentration of crystals for higher diffraction-pattern collection rates. *N*,*N*′,*N*′′-Triacetylchitotriose {*O*-[2-Acetamido-2-deoxy-β-d-glucopyranosyl-(1→4)]-*O*-[2-acetamido-2-deoxy-β-d-glucopyranosyl-(1→4)]-2-acetamido-2-deoxy-d-glucopyranose; Dextra, England} was dissolved in water and mixed with 5 *M* NaCl solution and 50%(*w*/*v*) PEG 4000 to achieve a concentration of 19.9 m*M* CTO in 1 *M* NaCl and 15% PEG 4000 before mixing with the microcrystal suspension.

The serial crystallography experiment was performed on the P11 beamline at PETRA III (Burkhardt *et al.*, 2016 ▸) using 13.5 keV photon energy X-rays focused to a spot of 4 × 8 µm (width × height) with a flux of 1.6 × 10^13^ photons s^−1^. A rotating beam chopper made of a 4 mm thick brass plate with holes for the X-rays to pass through was placed upstream of the focusing optics to generate X-ray pulses of 7.5 ms duration at a frequency of 25 Hz. The signal from a photodiode placed downstream of the chopper was used to trigger the readout of a PILATUS 6M detector, resulting in the collection of one diffraction image per pulse. The chopper was necessary to avoid sample heating caused by continuous exposure of the buffer to the intense focus and to allow control of the exposure time independent of sample delivery.

**Figure 1:**
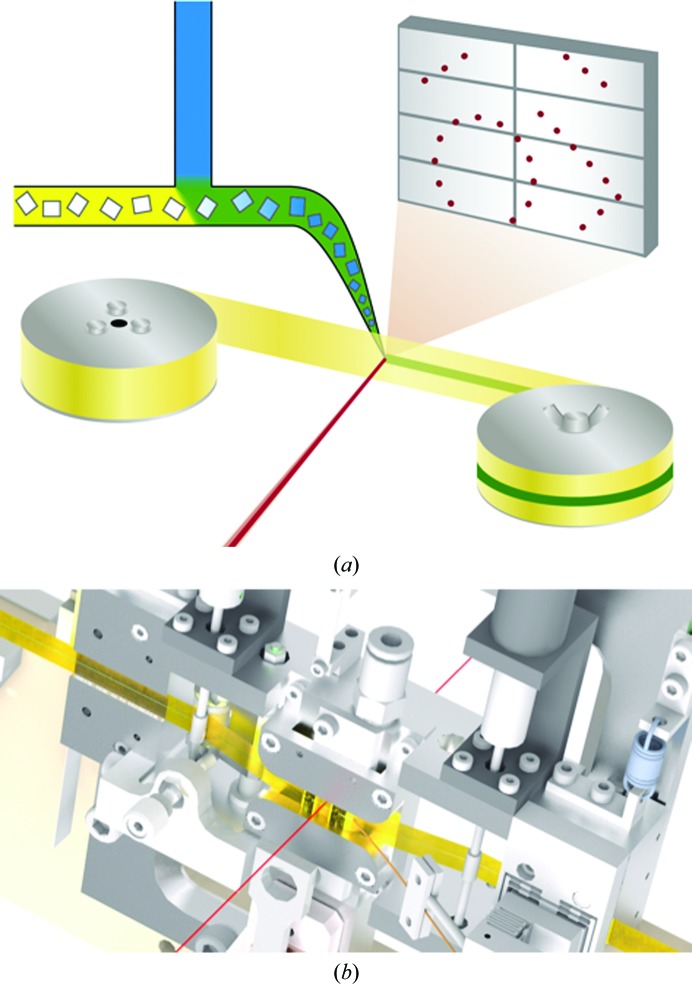
Schematics of the experimental setup. (*a*) The native protein crystal suspension (yellow) is mixed inline with the substrate solution (blue) before being deposited onto the detector side of the tape. The X-ray beam (red) is focused on the centre of the polyimide tape (yellow ribbon), which is being drawn from the feeder roll on the left to the collector roll on the right. A diffraction pattern measured in transmission is illustrated as red dots on the grey detector. (*b*) A technical drawing of the tape-drive device shows how tape is held under tension between two rollers near the interaction region and that X-rays pass through a hole in the centre of the device.

Sample delivery of the microcrystals to the X-ray focus was performed using a specially developed tape-drive device. As shown in Fig. 1 ▸, the lysozyme microcrystal suspension was deposited onto the surface of polyimide tape that was continuously drawn from a feeder roll to a collector roll. The movement of the tape under the sample capillary formed a stream on the tape that was aligned with the X-ray focus. A set of rollers and motors controlled the tape speed and kept the tape under slight tension. During the experiment, a constant tape speed of 0.6 mm s^−1^ was used. This speed was chosen as it resulted in a translation distance of 24 µm between frames, which was fast enough to avoid repeated crystal exposures. As-received rolls of nonsticky polyimide tape with a width of 6 mm and a thickness of 12 µm (Caplinq, The Netherlands) were directly mounted on the device. The tape position was vertically confined by grooves in the tape-drive body that matched the width of the tape, as shown in Fig. 1 ▸(*b*). A photograph of the setup during the experiment can be seen in Supplementary Fig. S1. Our tape-drive design was derived from a previous device used at the National Synchrotron Light Source (Roessler *et al.*, 2013 ▸) and LCLS (Roessler *et al.*, 2016 ▸), in which a tape conveyor belt transported acoustically ejected protein crystal suspension droplets of 10–100 µm in diameter to a microfocused X-ray beam. In contrast to this device, our tape was oriented such that the X-ray beam was incident normal to the tape. Using this configuration, we had a combined thickness of tape and sample solution of only around 50 µm, resulting in a low scattered X-ray background level.

The microcrystal suspension was placed in a reservoir that was attached to a motor programmed to rotate back and forth by 180° to prevent the protein crystals from settling during measurement. One side of the reservoir was connected to a water supply that was pressurized by an Elveflow OB1 flow controller to hydraulically actuate a plunger in the reservoir. The other side was connected to a borosilicate capillary with an internal diameter of 100 µm (Polymicro, USA), through which the microcrystal suspension flowed to a mixer before being deposited onto the tape. From a second reservoir, the aqueous ligand solution was also delivered to the mixer. Equal flow rates of microcrystal suspension and ligand solution of 0.6 µl min^−1^ were kept constant throughout the experiment, ensuring a 1:1 mixing ratio.

**Figure 2:**
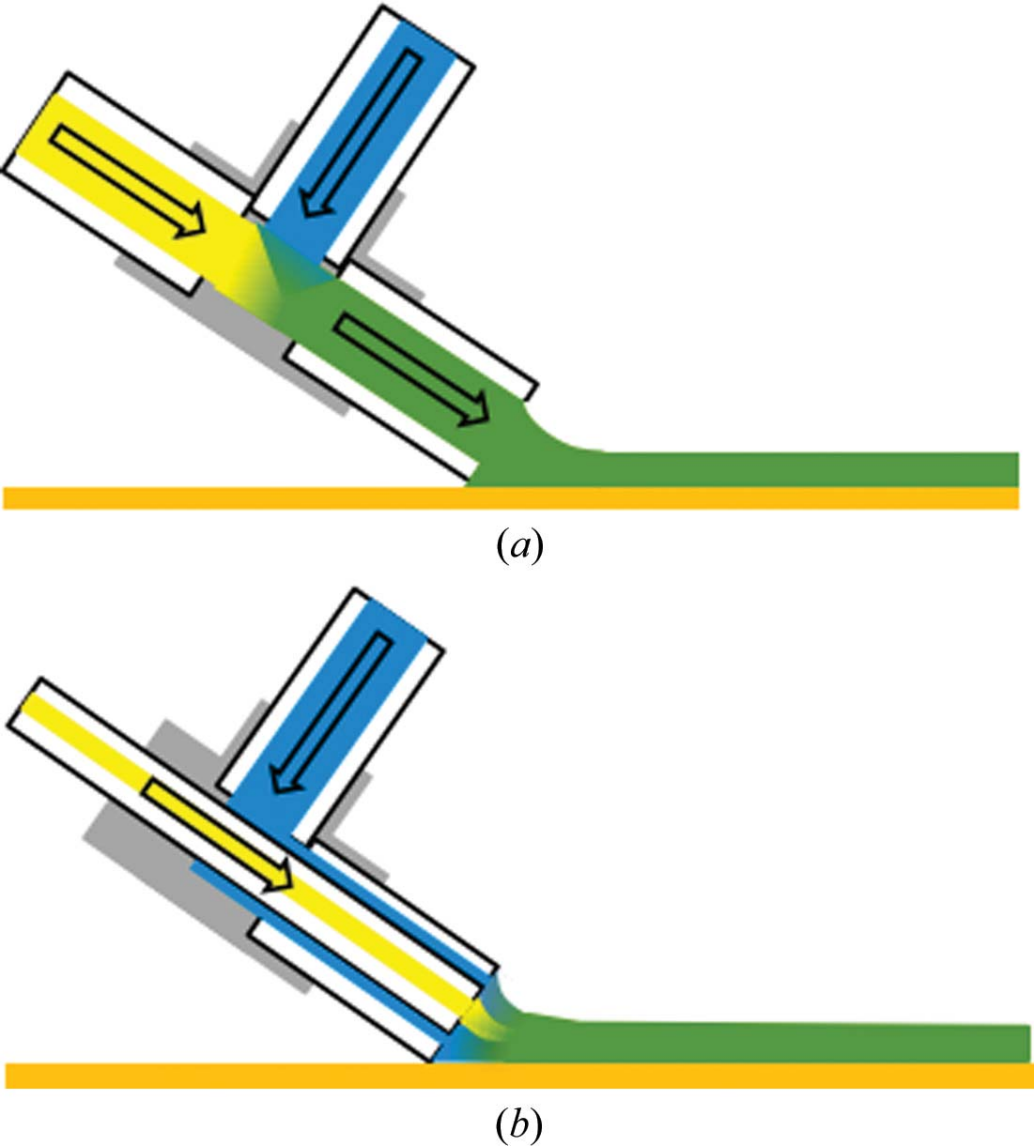
The mixer configurations for the (*a*) 50 s and (*b*) 2 s mixing times are illustrated, showing a side view of the sample-stream deposition on the tape (orange line). The colour scheme for the respective liquids is the same as in the experimental setup schematic (Fig. 1 ▸ *a*). The images are not to scale.

Two different mixing times, 2 and 50 s, were measured using different mixer configurations based on a three-port microfluidic T-junction (Upchurch). For the 50 s mixing time, mixing was performed by flowing crystal suspension and ligand solution into the T-junction and connecting an outlet capillary that transports the mixed solution to the tape (Fig. 2 ▸ *a*). The length of the outlet capillary was 120 mm, resulting in a 48 s transport time of the mixed solutions to the tape surface. The tip of the sample-delivery capillary contacted the tape about 1 mm away from the X-ray focus. The translation time of the crystals on the tape was then about 2 s, resulting in a total mixing time of 50 s. For the 2 s mixing time, the protein crystal and ligand solutions were mixed at the capillary–tape contact point. This was accomplished by feeding the crystal suspension capillary (inner diameter 75 µm, outer diameter 150 µm) through the T-junction and into a square capillary with an internal edge length of 150 µm (Fig. 2 ▸ *b*). The ligand solution then flowed around the crystal suspension capillary through the remaining space in the square capillary to mix with the protein solution on the tape. The tip of this concentric transport capillary was also positioned around 1 mm away from the X-ray focus, resulting in a mixing time of around 2 s.

Prior to the experiment, a series of tests were performed to understand the important parameters that determine the sample-stream width and thickness. Here, the width refers to the distance which the stream laterally spreads on the tape, while the thickness refers to the height of the stream normal to the tape surface. Assuming a micrometre focus and a frame rate of 25 Hz, tape speeds in the range of millimetres per second are sufficient to ensure that fresh sample is transported into the focus between frames. Capillaries of different sizes and contact angles were tested, but it was found that the sample-stream width for this slow tape speed was primarily determined by the sample flow rate and surface-wetting properties. A simple model assuming that the sample flow rate, *f*, is equal to the tape-translation speed, *v*, multiplied by the cross-sectional area of the stream was able to fit the dependence of the experimentally observed stream width on the flow rate and tape speed. Assuming a rectangular stream cross-section, the thickness of the sample stream is then given by *t* = *f*/(*vw*), where *w* represents the stream width. Using this expression and the observed width, streams of the lysozyme crystal suspension on untreated polyimide tape were found to keep an aspect ratio (*t*/*w*) of 0.08, consistent with a constant wetting angle. The total flow rate of 1.2 µl min^−1^ and tape speed of 0.6 mm s^−1^ used during the experiment resulted in a stream width of 500 µm measured by an inline microscope, which corresponds to a thickness of 40 µm.

The average time necessary for a ligand molecule to diffuse to a lysozyme binding site was substantially different for each of the mixer configurations. The diffusion time of a ligand molecule in solution, *t*_D_, can be calculated from the average diffusion distance, *x*, and diffusion coefficient, *D*, according to *t*_D_ = 〈*x*〉^2^/2*D*. The diffusion coefficient of chitotriose in water has been measured to be 3.5 × 10^−6^ cm^2^ s^−1^ (Groves *et al.*, 2004 ▸). Considering the 50 s mixing case, mixing was performed in the T-junction, so the longest diffusion distance is approximately the capillary internal diameter of 100 µm. This results in a maximum time of 14 s for chitotriose to diffuse in solution for this mixer. For the 2 s case, mixing is performed on the tape, where the concentric geometry of the capillaries should result in a layering of the solutions, as illustrated in Fig. 2 ▸(*b*). An estimate of the diffusion time is then found from half of the thickness of the protein crystal suspension layer, as the ligand is approaching the centre from all sides. We assume that the wicking and spreading of the solutions onto the tape quickly results in uniform layers. In this case, the relative thicknesses of the layers are given by the flow rates. Using the total stream thickness of 40 µm, and assuming ligand:crystal:ligand solution layer thicknesses of 10:20:10 µm, a maximum solution diffusion time of 150 ms is then found for this case. The diffusion time of chitotriose from the surface to the centre of a 10 µm lysozyme microcrystal should be around 5 ms (Schmidt, 2013 ▸). Therefore, for both mixer configurations diffusion through the solution is expected to take the most time. These diffusion times also serve as an estimate in the uncertainty in the starting time of the binding reaction for the two mixer configurations, as crystals at different positions in the stream would be exposed to the ligand molecules at different times in this range. In this regard, mixing on the tape allows a much more precisely defined reaction time.

Data were continuously collected for 8.5 h in each mixing configuration. This resulted in a total consumption of around 300 µl (18.9 mg) of lysozyme for each data set. The incoming data stream was monitored for hits using the *OnDA* software package (Mariani *et al.*, 2016 ▸) and the live hit rate was displayed for fast feedback during the experiment. An offline version of *OnDA* called *offDA* (Mariani *et al.*, 2016 ▸) was used to identify individual ‘hits’ from the complete set of collected diffraction patterns and convert them to HDF5 format. The detected hits were then indexed, integrated and merged in point group 4/*mmm* to a resolution cutoff of 1.70 Å using the *CrystFEL* analysis software for serial crystallographic data (White *et al.*, 2012 ▸, 2013 ▸, 2016 ▸; White, 2014 ▸).

MTZ files for crystallographic data processing were generated from *CrystFEL* merged reflection datafiles using *F*2*MTZ* from the *CCP*4 suite (Winn *et al.*, 2011 ▸). Figures of merit were calculated using *compare_hkl* (*R*_split_, CC_1/2_ and CC*) and *check_hkl* (SNR, multiplicity and completeness), which are both part of *CrystFEL*.

The lysozyme structure with PDB code 4et8 (Boutet *et al.*, 2012 ▸) was used as the starting model for both mixing cases. Owing to non-isomorphism of the collected data sets with that of 4et8, *R*_free_ flags were generated randomly using *phenix.refine* (Afonine *et al.*, 2012 ▸), and the same set of *R*_free_ flags was used for both data sets. Initial refinement was carried out using *phenix.refine*, with all isotropic atomic displacement parameters (ADPs) set to 20 Å^2^ and using simulated annealing. Ligands were then automatically placed in the initial *F*_o_ − *F*_c_ map using *LigandFit* (Terwilliger *et al.*, 2006 ▸, 2007 ▸) in *PHENIX* (Adams *et al.*, 2010 ▸), giving only the three-letter code CTO for *N*,*N*′,*N*′′-triacetylchitotriose as additional input. Restraints for CTO were generated using *ReadySet!* (Moriarty *et al.*, 2009 ▸) in *PHENIX*, followed by iterative cycles of restrained maximum-likelihood refinement using *phenix.refine* and manual model rebuilding using *Coot* (Emsley *et al.*, 2010 ▸). CTO grouped occupancy was refined in *phenix.refine*. After refinement, the isotropic ADPs of the tightest bound sugar ring of CTO matched the isotropic ADPs of the neighbouring lysozyme residues. Ordered solvent molecules and ions [chloride (Cl^−^) and sodium (Na^+^)] were placed in *Coot*, followed by further rounds of refinement in *phenix.refine* to convergence. *Polygon* (Urzhumtseva *et al.*, 2009 ▸) and *MolProbity* (Chen *et al.*, 2010 ▸) were used for validation of the final model and *ValLigURL* (Kleywegt & Harris, 2007 ▸) was used to check the geometry of the refined CTO molecule.

To assess the influence of the starting lysozyme model, structure refinement was also carried out in both mixing cases using *phenix.refine* starting with the structure of lysozyme co-crystallized with *N*,*N*′,*N*′′-triacetylchitotriose (PDB entry 1hew; Cheetham *et al.*, 1992 ▸). Before starting refinement, all isotropic ADPs were reset to 20 Å^2^, but no further modification of the structure was made as the ordered solvent and NAG-NAG-NAG bound to the active centre were unchanged. After initial refinement (rigid body, *xyz* coordinates, isotropic ADP, simulated annealing and real space), the resulting model and maps were inspected using *Coot*, followed by iterative cycles of restrained maximum-likelihood refinement using *phenix.refine* and manual model rebuilding using *Coot*. After the addition of riding H atoms with *ReadySet!* in *PHENIX*, the structural model was edited manually in *Coot* to remove the automatically placed, but chemically incorrect, H atom from the glycosidic O atom between C1 and C4 of the individual NAG residues. *Polygon* and *MolProbity* were used for validation of the final model. Final models from starting models based on both PDB entries 4et8 and 1hew for both mixing cases were compared with each other and with PDB entry 1hew in *PyMOL*.

## 3. Results   

A total of 147 407 and 142 265 indexable diffraction images were collected for the 2 and 50 s mixing times, respectively. This corresponded to 24% (2 s) and 27% (50 s) of the total collected detector exposures (‘indexing fraction’), resulting in an effective data-collection rate of 6–7 Hz (see Table 1 ▸ for details). Compared with the earlier serial synchrotron experiment by Stellato and coworkers, we have reduced the sample consumption drastically from 6.25 µg to 89 ng of protein consumed per indexable detector frame. This was mainly owing to the markedly lower sample flow rate of 600 nl min^−1^, combined with improvements in the data-analysis pipeline.

**Figure 3:**
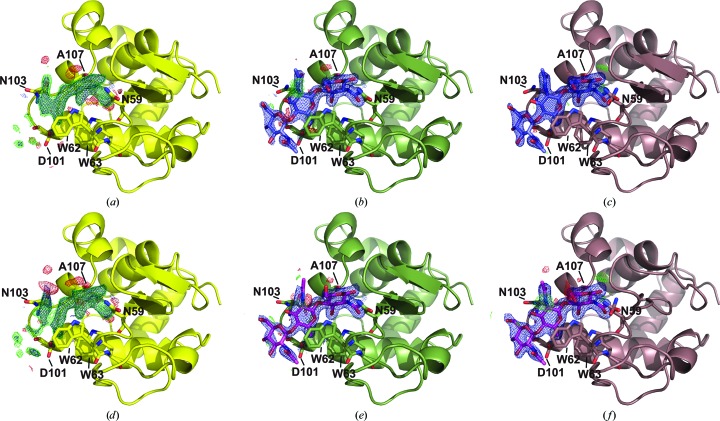
CTO bound to lysozyme: short-mixing case (*a*, *b*, *c*) and long-mixing case (*d*, *e*, *f*). (*a*) and (*d*) show the 2*F*_o_ − *F*_c_ (blue, 1σ) and *F*_o_ − *F*_c_ (green and red, 2.5σ) maps of the lysozyme binding site after initial refinement with ligand-free phases. (*b*) and (*e*) show the maps at the same levels after automatic ligand-placement and refinement, and (*c*) and (*f*) show the respective final refined models and maps. Residues forming hydrogen bonds to CTO are shown.

**Figure 4:**
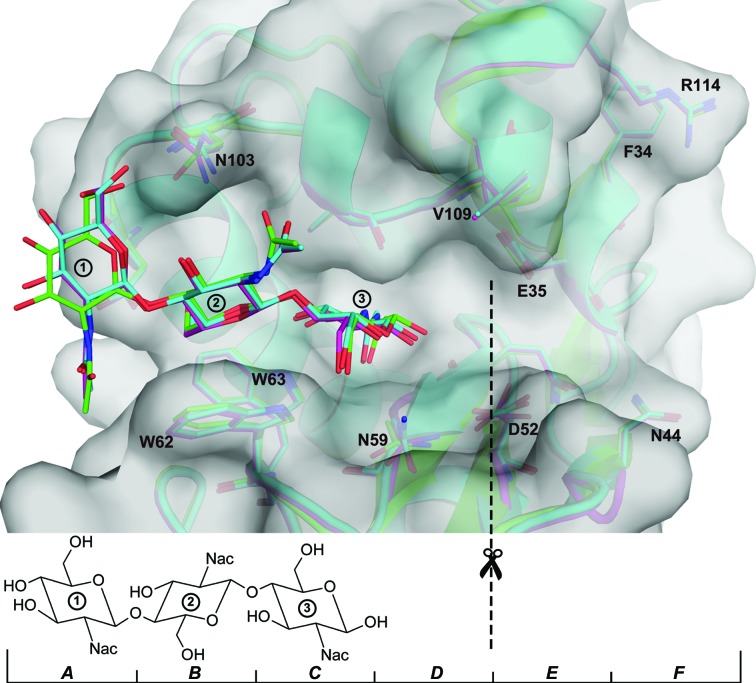
Comparison of ligand binding in the long-mixing case solved starting from PDB entries 4et8 (magenta) and 1hew (cyan) with that in the co-crystallized ligand structure PDB entry 1hew (green). It can be seen that all ligands bind to subsites *ABC* and that only the most flexible sugar ring 1 shows a significantly different orientation. Most notably, the different orientation of the hydroxyl O atom at position 6 of the pyranose ring and of the acetamido group of Asn103 results in a hydrogen bond between the ligand and protein in PDB entry 1hew but not in the mixing cases. Binding-site residues of lysozyme are displayed and the associated binding subsites *A*–*F* are indicated at the bottom of the figure. The dashed line indicates the active site for catalysis (cleavage site).

The electron-density maps obtained starting from the ligand-free model (PDB entry 4et8) described previously are shown in Fig. 3 ▸. In both mixing cases, the 2*F*_o_ − *F*_c_ and *F*_o_ − *F*_c_ maps reveal the presence of a large molecule near the active site. Phasing with a starting model containing 1→4 linked NAG-NAG-NAG (PDB entry 1hew) shows that the mixed-in ligand bound to subsites *A*, *B* and *C* in both cases (see Fig. 4 ▸ and Supplementary Fig. S2), which agrees with previous crystallographic (Cheetham *et al.*, 1992 ▸) and NMR (Raftery *et al.*, 1969 ▸) studies.

To minimize model bias, we based our refinement on the initial electron-density maps (see Figs. 3 ▸ *a* and 3 ▸ *d*) obtained from the ligand-free starting model PDB entry 4et8 (Boutet *et al.*, 2012 ▸). Using the *F*_o_ − *F*_c_ map, *LigandFit* from *PHENIX* (Terwilliger *et al.*, 2006 ▸, 2007 ▸) placed one molecule of CTO in the active site (see Figs. 3 ▸ *b* and 3 ▸ *e*). In both cases, the automatically placed orientation of the molecule agreed well with the known bound CTO positions in lysozyme (Cheetham *et al.*, 1992 ▸). Inspection of the map and model revealed only slight differences between the mixing cases, showing that a mixing time of only about 2 s is sufficient to populate 90% of the lysozyme active sites, found from grouped occupancy refinement. As reported in the literature (Cheetham *et al.*, 1992 ▸), the isotropic atomic displacement parameters of the atoms in the three NAG rings increased from the most tightly bound ring (close to binding site *C*) to the ring bound to binding site *A*. For the assessment of occupancy, we thus followed the rule that the isotropic ADP of the tightest bound NAG should correspond to that of the surrounding (binding) amino-acid residues. This resulted in CTO occupancies of 0.91 and 0.97 for the 2 and 50 s mixing times, respectively. The occupancy for the 50 s mixing time corresponds to that found in a bound single-crystal study (Cheetham *et al.*, 1992 ▸). Final rounds of refinement were carried out with these CTO occupancies; the resulting figures of merit are given in Table 1 ▸ and final maps and structural models can be seen in Figs. 3 ▸(*c*) and 3 ▸(*f*).

The overall root-mean-square deviation (r.m.s.d.) between the final refined models from both mixing times was 0.056 Å after alignment with *PyMOL*. The r.m.s.d.s between the starting model and final refined models were 0.152 Å (2 s) and 0.151 Å (50 s). Structural alignment with the model derived from co-crystallization of HEWL with CTO (PDB entry 1hew) gave r.m.s.d. values of 0.185 Å (2 s) and 0.189 Å (50 s).

Analysis of the hydrogen bonds between chitotriose and lysozyme using *PyMOL*, *PDBsum* (Laskowski, 2009 ▸) and *LigPlot*+ (Laskowski & Swindells, 2011 ▸) revealed that substrate binding occurred in both mixing cases to binding subsites *A*, *B* and *C*, in agreement with a previous study (Cheetham *et al.*, 1992 ▸). A comparison of the 50 s structural model with the 1hew model is shown in Fig. 4 ▸, while a comparison of the 2 and 50 s mixing cases is shown in Supplementary Fig. S2. In both structures sugar ring 1, located in binding subsite *A*, binds to lysozyme *via* a hydrogen bond between OD2 of Asp101 and the acetamido N atom in ring 1 (see Supplementary Figs. S3 and S4), while in the corresponding 1hew structure NAG1 binds to Asn103 through a hydrogen bond between OD1 and the hydroxyl O atom at position 6 of the pyranose ring and Asp101 only forms a hydrogen bond to NAG2. This corresponds to an almost identical bond between Asp101 in the 2 and 50 s mixing cases and the same hydroxyl O atom in ring 2 of CTO. The slight difference is that in the 2 s mixing case Asn103 is connected to CTO *via* a bound water molecule, whilst in the 50 s mixing case this water is missing and Asn103 only interacts with CTO through van der Waals forces.

Analysis of the extended solvent network between the bound ligand and lysozyme shows that for the 50 s mixing time four ordered solvent molecules are involved in binding (see Supplementary Fig. S3): three of these (O148, O170 and O180) directly form hydrogen bonds to CTO, while the fourth (O150) is connected to CTO through a hydrogen bond to O180. This fourth water stabilizes substrate binding through hydrogen bonds to Ile98, Asn103 and Gly104. O170 connects CTO to Gln57 and one of the alternate conformers of Asp52; together with Glu35 the latter forms the catalytic site of HEWL. O148 is connected to O170 and Ala107, both of which form hydrogen bonds to CTO and to Val109, which is part of binding subsite *D* (see Fig. 3 ▸). The water network of the 2 s case is far more extended (see Supplementary Fig. 4 ▸), especially along the cleft that forms the catalytic site. In the 50 s case only two waters adhere directly or indirectly (through other water molecules) to CTO along this cleft, whilst a total of eight water molecules (O151, O152, O166, O167, O171, O174, O194 and O210) form the extended solvent network in the 2 s case (see Supplementary Fig. 4 ▸). These ordered solvent molecules connect CTO to residues Phe34, Glu35, Asp52, Gln57, Ala107 and Val109, which are all part of the sugar-binding cleft (subsites *C*, *D* and *E*). Three additional solvent molecules connect CTO to residues Ile98, Asp101, Asn103 and Gly104, such that a total of 11 water molecules are involved in CTO binding in the 2 s case, as opposed to four water molecules in the 50 s case.

Contrary to our findings, a powder diffraction study found that CTO preferentially binds to subsites *B*, *C* and *D* (*BCD*; Von Dreele, 2005 ▸). The authors attributed the difference from previous crystallographic studies to the fact that CTO bound to positions *BCD* is hydrolyzed much faster than CTO bound to positions *A*, *B* and *C* (*ABC*). Thus, only CTO bound to subsites *ABC* is visible in crystallographic studies because these experiments take much longer from crystallization to data collection. In our experiment, we used a microcrystalline slurry and the time between mixing in CTO to probing the sample with X-rays was only a few seconds, as opposed to several minutes in the powder diffraction study. If binding of CTO to the *BCD* sites were indeed faster than to the *ABC* sites, we would expect to observe a mixture of both binding modes. As already discussed, we observed a clear preferred binding of CTO to subsites *ABC*. The 2*mF*_o_ − *DF*_c_ and *mF*_o_ − *DF*_c_ maps shown in Supplementary Fig. S5 contain only weak features at the *BCD* subsites at σ levels below 0.6 and 2.5, respectively. Furthermore, refinement starting from the powder diffraction structure (PDB entry 1sf6; Von Dreele, 2005 ▸) results in a model with a clear preference for the *ABC* binding mode. Supplementary Fig. S5 shows positive difference electron density at the position of sugar ring 1 of CTO from our model (bound to subsite *A*) and almost no electron density around the NAG molecule that would correspond to binding to subsite *D*.

## 4. Conclusion   

We have demonstrated that serial synchrotron crystallography can be used for mix-and-diffuse studies of ligand binding just 2 s after the ligand is mixed inline with the protein microcrystal suspension. The small lysozyme crystal size in our experiment resulted in diffusion times that were sufficiently short that nearly full occupancy of the bound substrate could be achieved in this time. This overcomes many technological obstacles to native protein drug screening, and also opens up the possibility of investigating enzyme structural dynamics on a similar timescale. Further advances that may reduce the total collection time of a data set to seconds are on the horizon. Detectors are becoming available that are able to collect thousands of frames per second (Henrich *et al.*, 2011 ▸; Pennicard *et al.*, 2013 ▸; Redford *et al.*, 2016 ▸), which could be combined with higher speed crystal delivery at brighter diffraction-limited storage rings (Eriksson *et al.*, 2014 ▸). Recently, it was shown that by using the high flux of nonmonochromatic ‘pink-beam’ undulator radiation at a synchrotron facility, single patterns from protein microcrystals could be acquired from 100 ps X-ray pulses arising from single electron bunches (Meents *et al.*, 2017 ▸). The advantage of using the broader bandwidth was demonstrated by the requirement for only 50 such exposures to generate a full diffraction data set.

## Supplementary Material

PDB reference: lysozyme, complex with chitotriose, 2 s mixing time, phased using PDB entry 1hew, 5njp

PDB reference: phased using PDB entry 4et8, 5njq

PDB reference: 50 s mixing time, phased using PDB entry 4et8, 5njr

PDB reference: phased using PDB entry 1hew, 5njs

Supplementary Figures S1-S5.. DOI: 10.1107/S2052252517013124/ec5004sup1.pdf

## Figures and Tables

**Table:** (*a*) Data collection.

	50 s	2 s
Temperature (K)	293	293
Crystal size (µm)	6–8	6–8
No. of collected images	527453	611182
Total measuring time (s)	30240	30480
Average acquisition rate (frames s^−1^)	25	25
No. of hits	169021	205181
Indexed patterns	142265	147407
Space group	*P*4_3_2_1_2	*P*4_3_2_1_2
Unit-cell parameters		
*a* = *b* (Å)	79.61	79.61
*c* (Å)	38.32	38.32
α = β = γ (°)	90	90
Resolution (Å)	22.68–1.70 (1.761–1.70)	22.68–1.70 (1.761–1.70)
〈*I*/σ(*I*)〉	16.65 (1.06)	14.53 (0.56)
Completeness (%)	100 (100)	100 (100)
Multiplicity	4932 (267)	3743 (165)
*R*_split_	3.49 (112.45)	4.06 (215.9)
CC_1/2_	0.99 (0.39)	0.99 (0.14)
CC*	0.99 (0.75)	0.99 (0.50)
Wilson *B* factor (Å^2^)	25.77	29.12

**Table:** (*b*) Refinement.

	50 s		2 s	
	4et8 phased	1hew phased	4et8 phased	1hew phased
PDB code	5njr	5njs	5njq	5njp
Resolution (Å)	22.68–1.70 (1.76–1.70)	22.68–1.70 (1.76–1.70)	22.68–1.70 (1.76–1.70)	21.81–1.70 (1.76–1.70)
No. of reflections	14040 (1352)	14040 (1352)	14017 (1339)	14038 (1339)
Reflections used for *R*_free_	1403 (136)	1403 (136)	1401 (134)	1401 (134)
*R*_work_	0.1661 (0.3175)	0.1641 (0.3185)	0.1631 (0.3644)	0.1702 (0.3615)
*R*_free_	0.1975 (0.3670)	0.1988 (0.3696)	0.2003 (0.3596)	0.2009 (0.3524)
No. of atoms				
Protein	1046	1001	1033	1009
Ligand/ion	47	47	46	46
Water	59	60	82	60
*B* factors (Å^2^)				
Protein	33.13	34.96	35.66	37.69
Ligand/ion	54.47	58.59	52.73	52.90
Water	51.37	41.37	45.58	44.43
Ramachandran favoured (%)	96.85	97.64	98.43	97.64
Ramachandran allowed (%)	3.15	1.57	1.57	2.36
Ramachandran outliers (%)	0	0.79	0	0
R.m.s. deviations				
Bond lengths (Å)	0.004	0.004	0.005	0.004
Bond angles (°)	0.64	0.62	0.70	0.64
Clashscore	0.93	0.00	1.41	2.43
