# Supplementary material for: Mix-and-diffuse serial synchrotron crystallography
Source: IUCrJ. 2017 Oct 9;4(Pt 6):769–77. doi: 10.1107/S2052252517013124 (PMC5668862; doi:10.1107/S2052252517013124)
Supplement: Supplementary file 1 [file m-04-00769-sup1.pdf]

IUCrJ

Volume 4 (2017)

Supporting information for article:

Mix-and-diffuse serial synchrotron crystallography

Kenneth R. Beyerlein, Dennis Dierksmeyer, Valerio Mariani, Manuela Kuhn, Iosifina Sarrou, Angelica Ottaviano, Salah Awel, Juraj Knoska, Silje Fuglerud, Olof Jönsson, Stephan Stern, Max Wiedorn, Oleksandr Yefanov, Luigi Adriano, Richard Bean, Anja Burkhardt, Pontus Fischer, Michael Heymann, Daniel A. Horke, Katharina E. J. Jungnickel, Elena Kovaleva, Olga Lorbeer, Markus Metz, Jan Meyer, Andrew Morgan, Kanupriya Pande, Saravanan Panneerselvam, Carolin Seuring, Aleksandra Tolstikova, Julia Lieske, Steve Aplin, Manfred Roessle, Thomas A. White, Henry N. Chapman, Alke Meents and Dominik Oberthuer

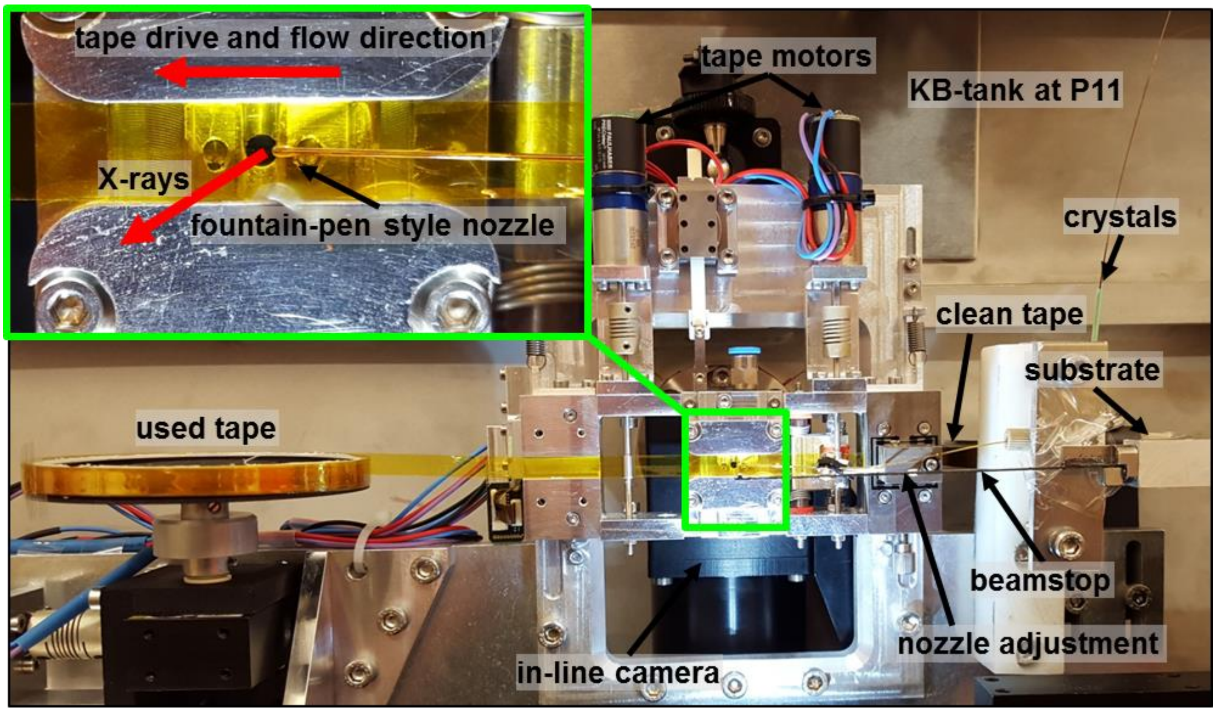

Figure S1 Tape drive setup as mounted at beamline P11 (PETRAIII/DESY).

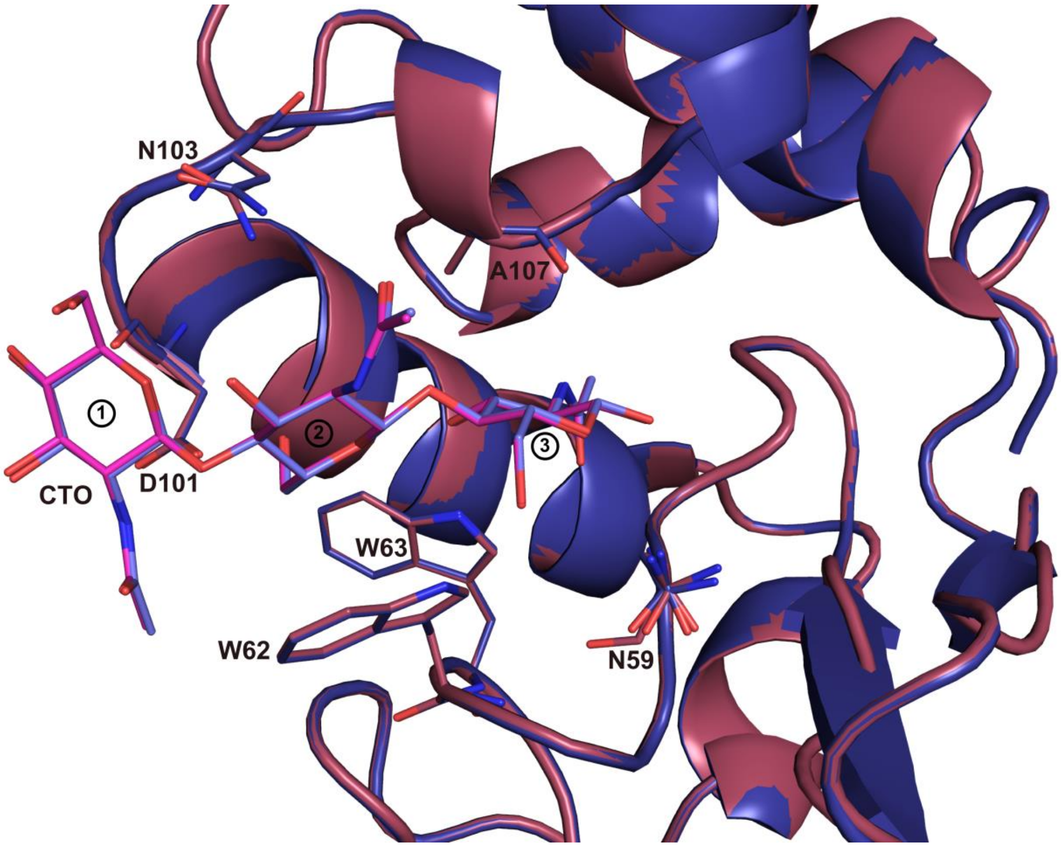

Figure S2 Comparison of the final refined structural models of the 50s (red/magenta) and 2s (purple) mixing case. Protein residues shown as sticks all form hydrogen bonds with CTO.

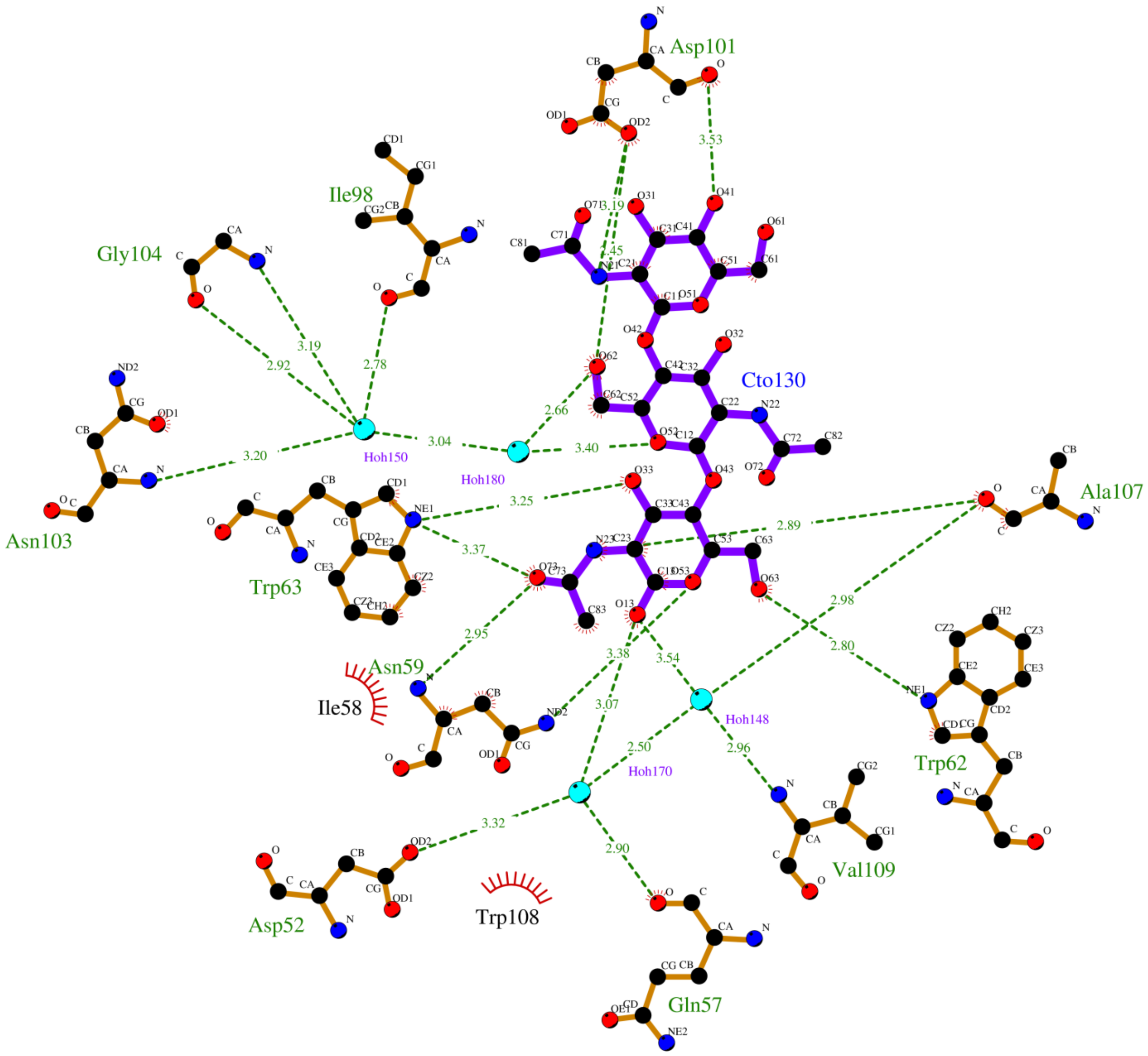

Figure S3 LigPlot of the binding of CTO in the 50 seconds mixing case. Lysozyme residues that form hydrogen bonds (dashed green lines) with CTO or with the water molecules of the extended CTO-water network are shown. The numbers in the dashed green lines indicate the interatomic distances as determined by Coot and LigPlot+.

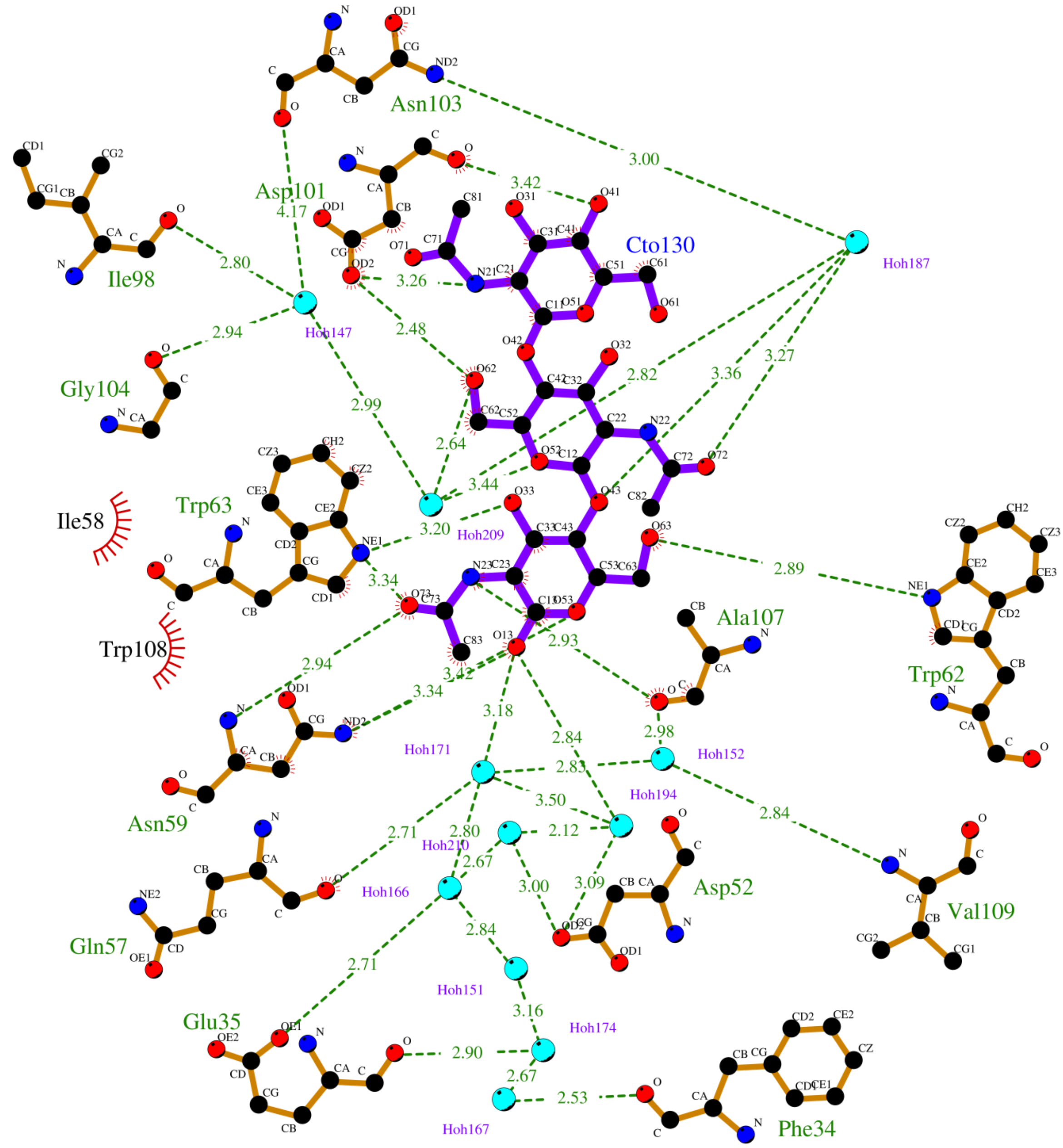

Figure S4 LigPlot of the binding of CTO in the 2 seconds mixing case. Lysozyme residues that form hydrogen bonds (dashed green lines) with CTO or with the water molecules of the extended CTO-water network are shown. The numbers in the dashed green lines indicate the interatomic distances as determined by Coot and LigPlot+.

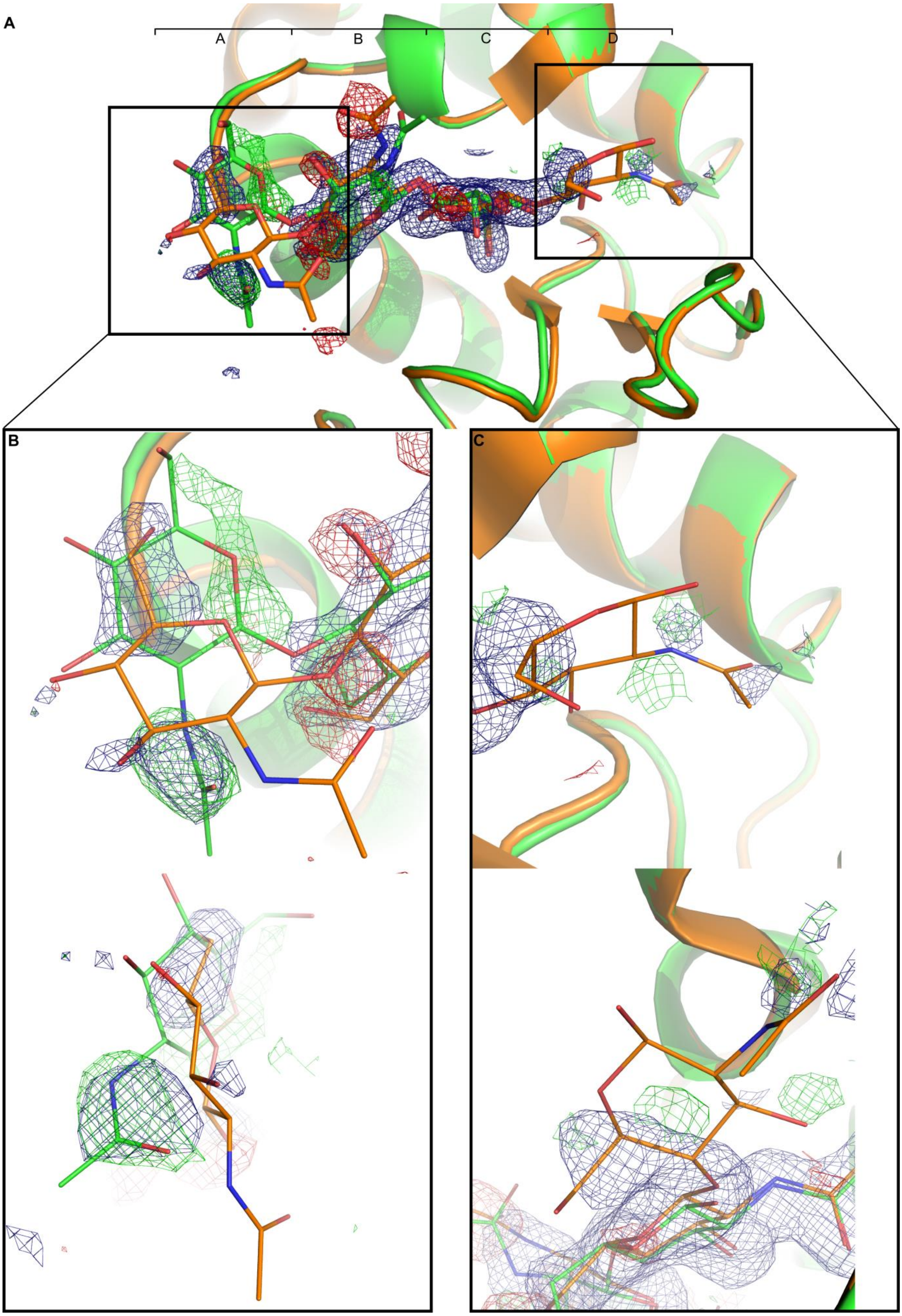

Figure S5 A) The structural model from a powder diffraction study (PDB-code: 1SF6) was refined against the data of the 50s mixing case. The resulting 2mFo-DFc map at 1σ and mFo-DFc (at 2.5 σ)

map are shown over the resulting structural model (orange) and the final model of the 50s mixing case (green). In B) the sugar rings of CTO bound to subsite A are shown from two different angles. Almost no electron density can be seen around ring 1 of the model (orange) that resulted from refinement of the model deposited under 1SF6 with our data, while there are strong map features around the position of ring 1 of CTO of the final refined model (green). In C) the third sugar ring (orange, at binding site D) corresponding to binding mode BCD is displayed in detail from two different viewing positions. Almost no electron density is visible around most of this ring.
